# COVID‐19 and protected areas: Impacts, conflicts, and possible management solutions

**DOI:** 10.1111/conl.12800

**Published:** 2021-04-06

**Authors:** Nikoleta Jones, James McGinlay, Angela Jones, Chrisovalantis Malesios, Jens Holtvoeth, Panayiotis G. Dimitrakopoulos, Vassilis Gkoumas, Andreas Kontoleon

**Affiliations:** ^1^ Department of Land Economy, University of Cambridge Conservation Research Institute University of Cambridge Cambridge UK; ^2^ Snowdonia National Park Authority Penrhyndeudraeth Wales UK; ^3^ Department of Environment University of the Aegean Mytilene Greece

**Keywords:** biodiversity conservation, lockdown, overcrowding, protected areas management, social impacts, visitors, Wales

## Abstract

During the first wave of the COVID‐19 pandemic, management authorities of numerous Protected Areas (PAs) had to discourage visitors from accessing them in order to reduce the virus transmission rate and protect local communities. This resulted in social–ecological impacts and added another layer of complexity to managing PAs. This paper presents the results of a survey in Snowdonia National Park capturing the views of over 700 local residents on the impacts of COVID‐19 restrictions and possible scenarios and tools for managing tourist numbers. Lower visitor numbers were seen in a broadly positive way by a significant number of respondents while benefit sharing issues from tourism also emerged. Most preferred options to manage overcrowding were restricting access to certain paths, the development of mobile applications to alert people to overcrowding and reporting irresponsible behavior. Our findings are useful for PA managers and local communities currently developing post‐COVID‐19 recovery strategies.

## 1 INTRODUCTION

In response to the COVID‐19 pandemic, many management authorities of Protected Areas (PAs) discouraged visitors from accessing them in order to slow down the transmission rate and protect local communities (IUCN, 2020; Jacobs et al., 2020; McGinlay et al., 2020). As a consequence, the COVID‐19 pandemic is expected to have significant socioeconomic implications for local communities living near or inside these areas (Bennett et al., 2020; Hockings et al., 2020). Currently there is limited empirical evidence on how COVID‐19 restrictions have impacted the everyday lives of local communities whose wellbeing is directly dependent on accessing PAs (Buckley et al., 2019; Naidoo et al., 2019; Romagosa et al., 2015). Gathering of such evidence will assist management authorities across the world to capture the complexities of these new challenges and plan actions for the future.

In this paper, we present the results of a survey which was distributed in June 2020 in Snowdonia National Park (NP), Wales, UK. Strict restrictions were imposed locally and nationally during Spring 2020 limiting both visitors and locals from accessing the most heavily used areas of the NP. Our study, reporting the views of over 700 local residents, provides evidence of how the restrictions impacted local communities, people's opinions on possible options for managing the PA during the pandemic and management tools to deal with high numbers of visitors. A key aim of the survey was to provide management authorities with data that would be used in decision‐making processes regarding both the management of PAs during the pandemic and for the development of post‐COVID‐19 recovery strategies.

## 2 METHODS

### 2.1 Research area

Snowdonia (Eryri) was designated as a National Park in 1951 and covers 2,132 square km. The NP comprises a diverse mix of landscapes and it is well known for its mountain ranges. It is is home to approximately 26,000 people. In 2018, Snowdonia NP experienced its highest recorded visitor figures of 4.48 million (STEAM data, 2018). Consequently, the local economy relies significantly on tourism and recreational activities. On March 23, 2020, new regulations came into force in Wales limiting people's movement and everyday activities to curb the spread of COVID‐19. Emergency legislation restricted access to the most popular spots in PAs both for visitors and locals, which remained in place for a period of 15 weeks. Snowdonia experienced the highest annual number of day visitors just before these official lockdown regulations were announced.

### 2.2 Questionnaire description

A structured questionnaire was developed in order to explore: (a) the socioeconomic impacts of COVID‐19 restrictions for local communities living near or inside the NP and (b) options to manage the park during the pandemic. The survey was part of a wider research project (FIDELIO) capturing social impacts of European protected areas and the impact of the pandemic for local communities living within them (https://www.fidelio.landecon.cam.ac.uk). Regarding the impact of COVID‐19 restrictions on accessing the NP a number of issues were explored influenced by previous studies (Bennett et al., 2019; Jones et al., 2020; Oldekop et al., 2016). These included change in recreational activities, quality of life, social relations with locals, connectedness to nature, mental and physical health, and personal income. All impacts were assessed using 5‐point Likert scales (Bennett et al., 2020) with higher values representing the most positive level of perceived impact, lower values the most negative level and the middle value representing a neutral evaluation (Table S3). Studies capturing people's perceptions of PAs have significantly increased in the conservation literature (Ban et al., 2019; Bennett, 2016; de Lange et al., 2016) allowing a subjective assessment of numerous social issues such as wellbeing, culture, conflicts, and social relations (de Lange et al., 2016).

Regarding management options during the pandemic (with a specific focus on the early months after the strict lockdown), respondents were presented with four hypothetical scenarios (Table 1). Participants were also asked to explain the reasons they agreed with the different options in an open‐ended format. Preferences for a number of more specific policy tools in order to manage overcrowding and irresponsible behavior were also explored.

**TABLE 1:** Options for managing visitors numbers

Management option	Description	Measurement scale
National Park open to everyone	Allow unrestricted access to everyone	5‐point Likert scale (1 totally disagree, 5 totally agree)
Spatially Phased Reopening	Phased reopening of areas and facilities, from the least visited and where spread of the virus was low risk, to the most visited/popular areas and facilities where spread of the virus was higher risk	
Restrict the number of out of area users	Allowing access to local residents and only to a limited number of out of area users every day	
Restrictions for all out of area users	Access would be allowed to the National Park only for people living locally until it would be considered safe to allow visitors back in the area.	

### 2.3 Sample

The questionnaire was distributed online in June 2020 using Qualtrics. The research team sent 3,000 postcards to a randomly selected sample of households in the area inviting them to access the survey via an online link. This was estimated to be just over 10% of the total population. The survey was also advertised online via social media and informal networks. In total 740 responses were included in the final analysis after excluding entries with missing values and responses from out of area users. The demographic characteristics of the final sample are presented in Table S1. When comparing the characteristics of the sample with those of the actual population there is a higher proportion of people who have completed degrees of higher education. Also, people over 75 years of age are slightly underrepresented in our sample. These limitations are likely to be due to the online distribution of the questionnaire because of COVID‐19 restrictions. The demographic items utilized in the statistical analysis as explanatory variables in regression modeling include an adequate number of responses in all categories, ensuring a robust statistical inference and estimation.

### 2.4 Data analysis

The data collected were analyzed using SPSS 26.0. Open‐ended answers were analyzed with N‐Vivo 12.0 capturing key themes in the participants’ comments (QSR, 2019). In order to explore people's views on the management options, an ordinal regression was applied (logit link function) including a number of explanatory variables (Agresti, 2010). Α backward elimination variable selection approach was applied to obtain the best fitted model to the data, starting by including all variables and removing at each step ones that were less statistically significant (Jones et al., 2015). To further avoid multicollinearity effects, variables measuring similar topics were merged into factors via a Principal Component Analysis (PCA). In particular, explanatory variables introduced in the regression models were the following:

(A) The impact of lockdown restrictions on everyday life. The initial 9 variables measuring this aspect were reduced to 3 new factors via PCA (Table S2):

A1. Factor A1 included variables capturing the impact from the reduced number of visitors in outdoor spaces (quieter walking paths, cycling routes, and fewer visitors in beauty spots)
A2. Factor A2 included variables measuring the impact of lockdown on activities linked with social contact (shop closures, keeping 2 m distance and socializing with other people)
A3. Factor A3 included variables measuring impact of lockdown restrictions on aspects inside people's households (working from home, spending more time with household members and less travel).

(B) Impact of restrictions regarding the use of the NP. One new factor was created including all variables measuring these impacts using a PCA (Table S2) (merged variables: impact on quality of life, mental and physical health, connectedness to nature, social relations, and participation in recreational activities).
(C) The impact of lockdown on income level (measured on a 5‐point Likert scale)
(D) Demographics including age (D1), education (D2), income (D3), and gender (D4) (Tables 3 and S1).

A detailed presentation of all initial explanatory variables in the regression model (factors A1, A2, A3, B, and variable C) is provided in Table S3.

## 3 RESULTS

### 3.1 Impacts of COVID‐19 restrictions

**FIGURE 1:**
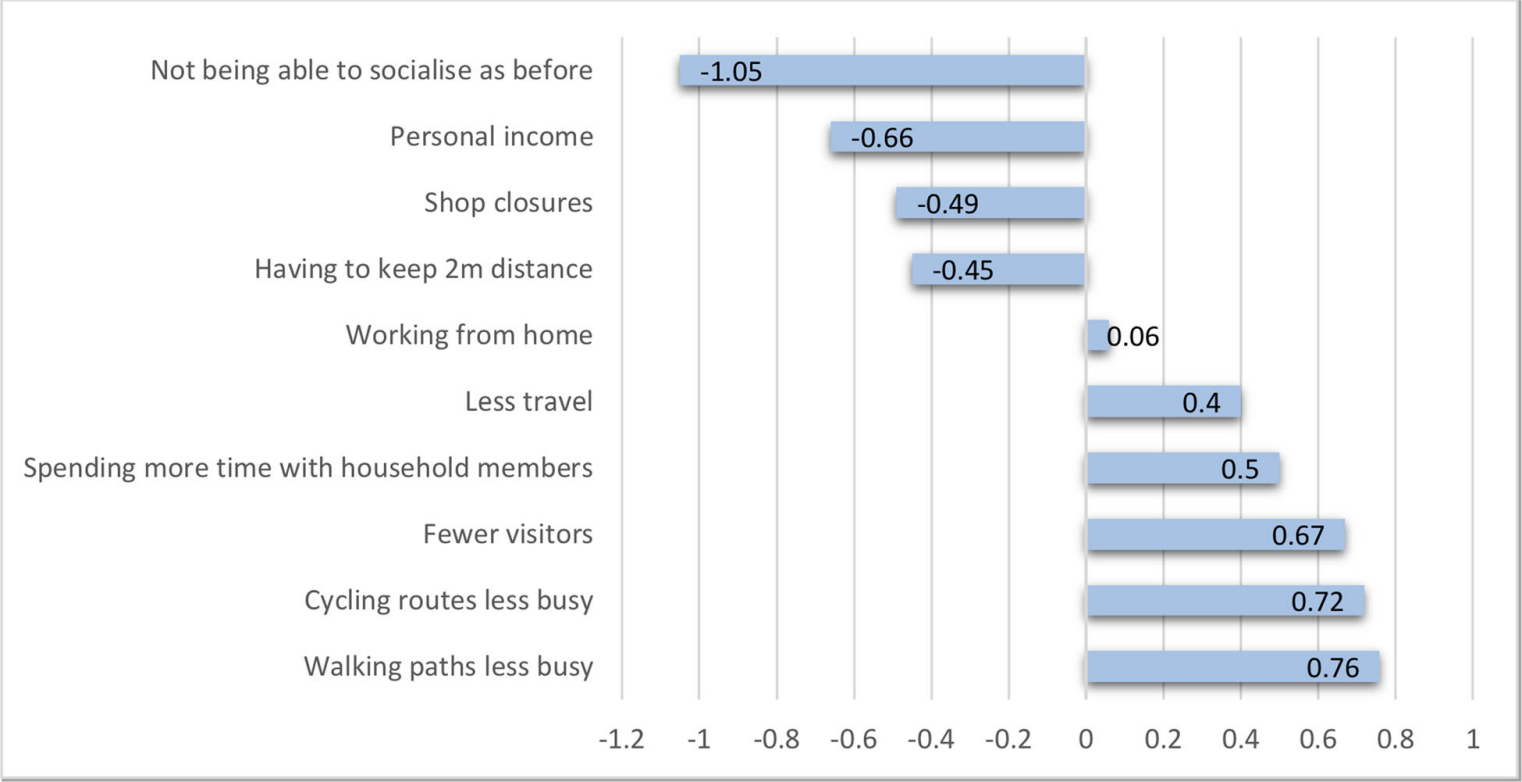
Life during lockdown for locals in Snowdonia National Park (Spring 2020): Positive and negative aspects

**FIGURE 2:**
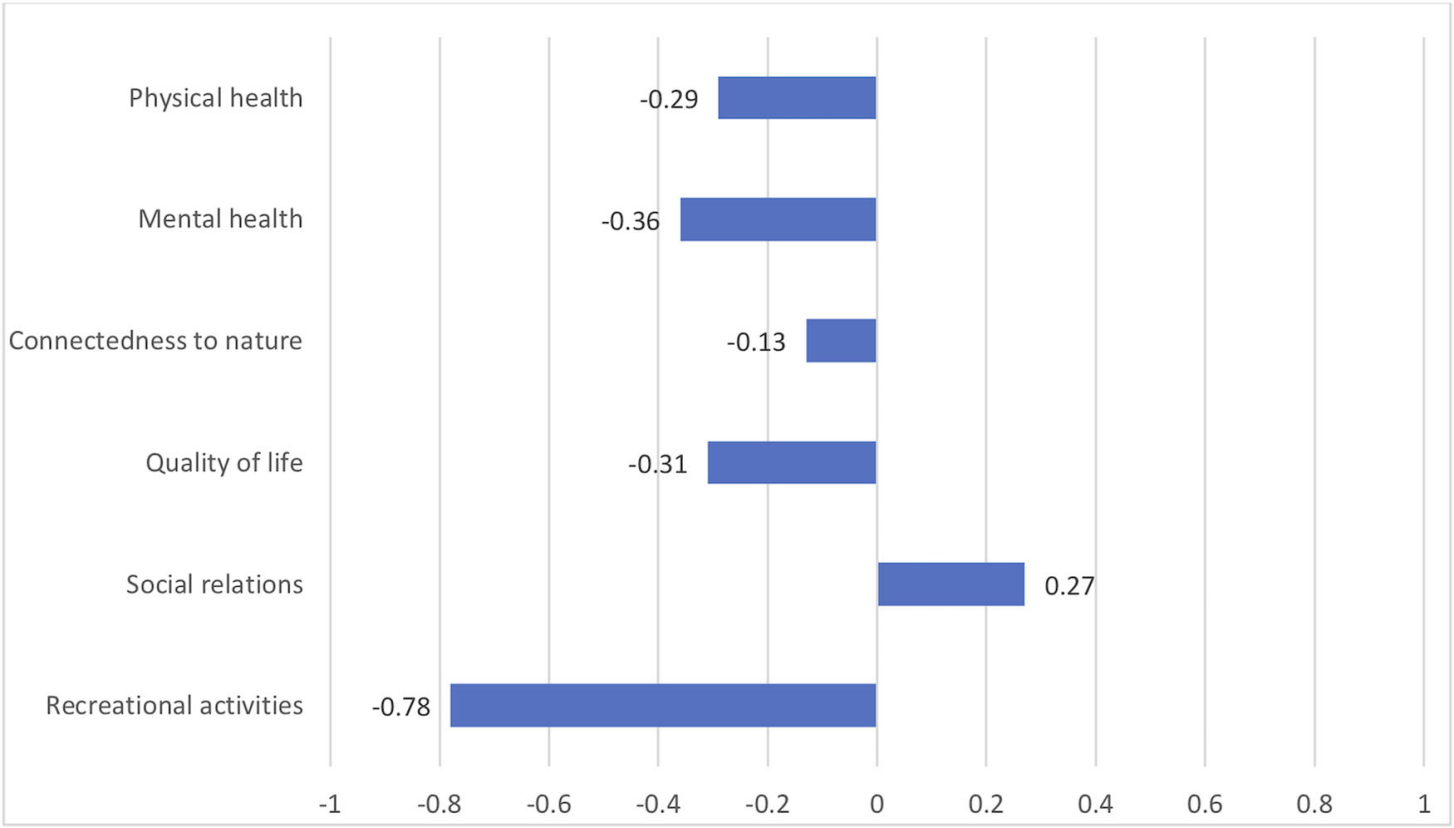
Impact of COVID‐19 restrictions linked with the National Park

The survey presented to respondents a range of potential practical changes or impacts of COVID‐19 restrictions. Respondents’ scores revealed both whether they had noticed such impacts, and their personal subjective evaluation of the impacts as positive, negative or neutral, which would be expected to vary from respondent to respondent, depending on personal circumstances. The baseline against which the new lockdown situation was judged would be expected to be the respondents’ subjective perception of the usual situation at the corresponding time the years prior to the survey (Figure 1). Respondents’ perceptions of the implications of the wide variety of changes caused by the lockdown on aspects of respondents’ well‐being were then further assessed in the survey (Figure 2).

Impacts of COVID‐19 restrictions that were evaluated by respondents as most positive were that walking and cycling paths were not as busy as usual and that the number of visitors had been reduced in areas of natural beauty. In contrast, most negatively evaluated impacts were on income and not being able to socialize with other people as before (Figure 1). Participants also evaluated that restrictions on accessing parts of the PA had a negative impact, especially on recreational activities, mental health and quality of life (Figure 2). Nevertheless, respondents evaluated that overall, social relations were improved during lockdown. However, this overall assessment masked a diversity of social impacts which were generally evaluated as both positive (spending more time with household members, increased use of the Welsh language) and negative (not being able to socialize as before, conflicts generated by residents not recognizing each other as local and therefore accusing them of being visitors).

### 3.2 Participants’ views on alternative management options during the pandemic

Four potential options were presented to respondents regarding the “reopening” of the Park and its management during the pandemic (Table 1). From the four options the preferred one was the (geographical) phased reopening of the NP followed by keeping restrictions for out of area users in the near future (June 2020) while the risk of transmission was considered high (Table 2).

**TABLE 2:** Preferences for different management options during COVID‐19

	Strongly disagree (%)	Somewhat disagree (%)	Neither agree nor disagree (%)	Somewhat agree (%)	Strongly agree (%)
National Park open to everyone	44.4	19.5	6.9	15.7	13.6
Spatially phased reopening	11.8	13.8	7.5	40.2	26.7
Restrict the number of out of area users	29.9	28.6	11.5	21.3	8.6
Restrictions for all out of area users	15.8	16.7	5.7	29.1	32.7

### 3.3 Factors explaining preferences for the management options

#### 3.3.1 Unrestricted access to all (full reopening to visitors)

A potential full reopening of the park to visitors was the least favored option. Regression analysis (Table 3) revealed that respondents who considered they had been positively impacted by lockdown restrictions tended to be more neutral or disagree with the full reopening of the park. Also, those whose income was not affected or was positively affected during lockdown tended to be less in favor of this option in comparison to the other respondents. Respondents below 65 years of age were also more willing to accept the full reopening of the park to visitors. In the open‐ended comments collected, this option was considered as the least complex as it doesn't involve any special interim management, just a return to normal but here responses were polarized between those emphasizing the economy and dependence on tourism, pushing for immediate or rapid reopening, and fear of COVID‐19 health impacts, favoring delay. Many respondents recognized the balance between the two so the only decision left was that of when exactly would be the right time to lift existing restrictions and move to a complete reopening.

**TABLE 3:** Results of ordinal regression explaining preferences for the different management options

			Restricted access to all out of area users		Limited access to visitors		Unrestricted access to all	
			Est	Wald	Est	Wald	Est	Wald
Dependent: Preference for management option	Strongly disagree		−1.59**	3.17	−1.48*	3.12	4.97***	84.97
	Disagree		−0.93	1.09	−0.68	0.66	5.59***	107.52
	neither disagree or agree		−0.78	0.77	−0.33	0.15	5.84***	117.17
	Agree		0.03	0.00	0.63	0.56	6.51***	145.00
	Reference category: Strongly agree							
Independent variables	A1. Impact of reduced visitor numbers		0.29***	26.05			−0.38***	42.01
	A2. Impact on social interactions		0.09*	3.04	−0.13**	6.49	−0.22***	15.38
	A3. Impact of staying at home		0.10***	3.52			−0.14***	5.82
	B. Impact of restrictions on visiting the National Park		0.12**	4.41	0.12**	4.35	−0.15***	6.02
	C. Impact of lockdown on income level	Very negative			0.59	1.41	−0.33	0.46
		Negative			0.97**	3.84	−0.61	1.58
		Neutral			0.92*	3.54	−0.80*	2.83
		Positive			1.15**	4.51	−1.45**	6.55
		Ref category: Very positive						
	D1. Age	18–25			−0.94	1.67	4.67***	112.90
		26–35			−1.30**	3.72	5.71***	641.58
		36–45			−1.36**	4.08	5.77***	651.25
		46–55			−1.42**	4.46	5.83***	722.10
		56–65			−1.50**	4.99	5.66***	678.85
		66–75			−1.20*	3.17	5.46	110.20
		Reference category: 76+						
	D3. Income	No income	0.67**	3.34				
		Up to £25,000	0.09	0.20				
		£25,001–50,000	−0.04	0.04				
		£50,000–70,000	0.08	0.17				
		Reference category:						
		Over £70,000						
	D4. Gender	Male			−0.32**	9.81		
		Ref category: Female						

* *p* < .1.

** *p* < .05.

*** *p* < .01.

#### 3.3.2 Spatially phased reopening

The results of the ordinal regression (Table 3) revealed no statistical significant parameters explaining preferences for the phased reopening of the NP. From the open‐ended comments, feasibility was the most common concern expressed by participants as it would be spatially relatively challenging to manage and respondents noted that it would require significant monitoring. Risk of confusion was another concern. As this option could be relatively complex, with some areas and facilities open and others not, several respondents felt that trying to communicate which areas are open would be challenging and might cause greater confusion. Furthermore, a large number of respondents felt that this option would not solve potential problems with overcrowding.

#### 3.3.3 Allowing access to local residents and only to a limited number of out of area users

The impact of lockdown restrictions on income levels explained to some extent preferences for this option with people who have not been heavily influenced by the lockdown (either negatively or positively) being more in favor (Table 3). Furthermore, those who considered that they were positively influenced by the restrictions in relation to the use of the NP were more supportive of this option. On the contrary, those who regarded that the impact of the lockdown on social relations was negative for them were less willing to accept this option. The results of the ordinal regression also revealed that male respondents and those who were between 26 and 75 years of age tended to disagree with this option. Main concerns expressed by participants about this proposed scenario were that it would be very difficult to enforce it and that it might also lead to divisions between groups of users. If such an option was adopted in the future then it would need careful design of new tools.

#### 3.3.4 Restricted access to all out of area users

Participants who considered that there were positive impacts from lockdown restrictions were also more in favor of this option (Table 3). These included impacts due to reduced number of visitors such as the quieter beauty spots, cycling, and walking paths. Also perceived impact on spending more time in one's house such as less travel, spending more time with household members and working from home were also significant explanatory parameters. Those who did not perceive there were negative impacts due to the lack of socializing had more positive views toward this option. Similarly, participants who considered that restrictions in relation to the use of the NP were positive were also more positive and preferred that restrictions for out of area users remain. Key issues emerging from the open‐ended questions were the difficulty of defining “locals,” the fact that since Snowdonia is a national park it should be open for everyone and fear of conflicts emerging between different users.

### 3.4 Tools to manage overcrowding

**FIGURE 3:**
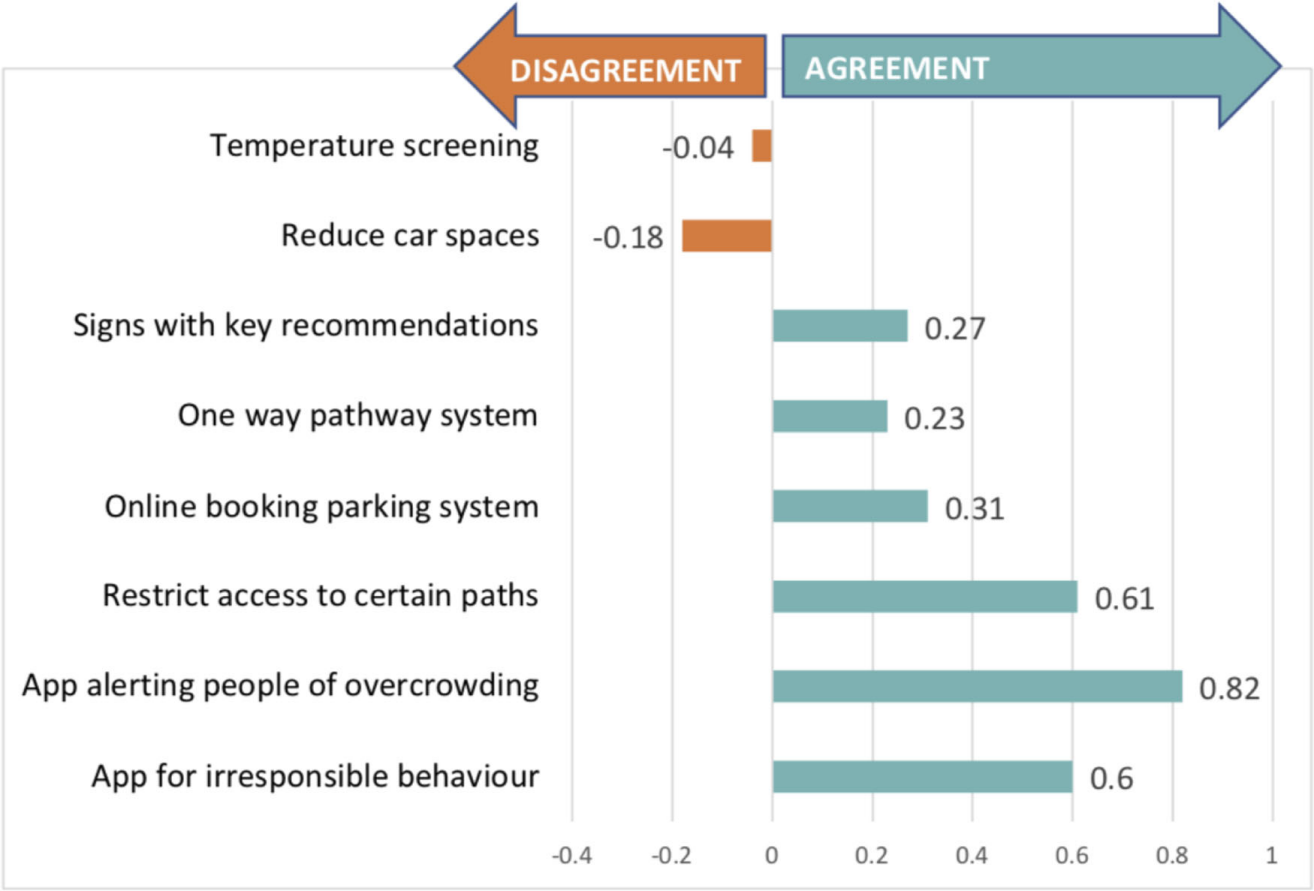
Preferences for different tools managing overcrowding in Snowdonia National Park

Participants were also asked to state their preference for a number of tools that could assist in managing overcrowding during the pandemic, especially if the number of visitors had to be managed. Considering that Snowdonia often has a large number of visitors, some of these tools could be used also after the pandemic to control visitor numbers and to manage irresponsible behavior. Most favored options were the development of mobile applications that would alert people to overcrowding incidents and also an application that people could use in order to report irresponsible behavior. Restricting access to certain paths which potentially can become overcrowded was also favored by several respondents. The least favored options were temperature screening at certain locations of the NP and also reduction of car spaces in designated car parks (Figure 3).

## 4 DISCUSSION

The local social–economic system in Snowdonia relies significantly on tourism. As a result, lockdown restrictions imposed during Spring 2020 had significant negative economic impacts for local residents, especially those involved in tourism. Our study also shows that in most cases people's preferences for the gradual “reopening” of the park to out of area users were influenced by the impact they experienced from lockdown restrictions. Those whose life was not affected by restrictions were more in favor of tighter rules with strict management of visitors numbers or even complete exclusion from the NP until the risk was considered low.

This finding highlights the potential existence of social equity issues (Dawson et al., 2017; Zafra‐Calvo et al., 2019) that often emerge in PAs due to uneven benefit sharing from tourism (Heslinga et al., 2017). In Snowdonia, these issues were most likely further aggravated due to new restrictions on visitors accessing the area during the first wave of COVID‐19. An important question thus emerges on how new potential mechanisms can be developed for equal benefit sharing in the long term (Snyman & Bricker, 2019). Increasing adaptive comanagement (Islam et al., 2018) and coproduction of knowledge with local stakeholders (Christie et al., 2017) can be considered a first step in this direction.

Our findings also highlight that the reduction in visitors may have resulted in a positive experience for many residents in Snowdonia. As noted, overall social relations were evaluated by respondents to have improved during the first wave of COVID‐19. The evidence suggests two likely reasons for this: the reduced number of visitors to the NP and (less obviously, but suggested by respondents’ qualitative comments) a higher adaptive capacity of local communities in Snowdonia during such uncertain times due to the rural character and small settlement size of the area (de Luca et al., 2020).

The fact that lower visitor numbers were seen in a broadly positive way by a significant number of respondents suggests that local communities may perceive problems in normal times with overcrowding, inadequate infrastructure for visitors and perhaps even overtourism in the Park. There is also some early evidence that such problems were more frequent in PAs in the past eight months due to COVID‐19 (McGinlay et al., 2020). Thus a key question, irrespective of the pandemic, is whether new measures need to be introduced in areas such as Snowdonia to control the number of visitors in the long term. The tools explored in the study provide some first potential directions. However, such measures need to be carefully designed and coproduced with local communities (Christie et al., 2017) taking into consideration key principles of equitable governance, moving away from the current top‐down decision‐making system of COVID‐19 restrictions.

## 5 CONCLUSION

During the COVID‐19 pandemic management authorities of PAs across the world have to find ways to manage the number of visitors in order to keep people safe whilst protecting biodiversity and the quality of life of local residents. Our study in Snowdonia NP revealed that COVID‐19 had significant negative impacts on people's everyday lives in the area. However, it also gave the opportunity for locals to enjoy the NP with no visitors and strengthen social relations. This has caused the resurgence of a debate regarding an optimum visitor carrying capacity in PAs. The results of our study indicate that local users in Snowdonia could be open to a number of new tools for managing the number of visitors and allowing the socioeconomic recovery of local communities in a more sustainable way after the pandemic. Managing visitor numbers and promoting responsible behavior could have two long‐term benefits: an improved quality of life for locals and reduced pressure on ecosystems due to reduced pressure from human activities. However, the mosaic of different views amongst residents on how visitor numbers can be managed in Snowdonia NP highlights that any future tools, both during and after the pandemic, need to take into account issues of social equity and consider the views of the public when developing post‐COVID‐19 recovery strategies.

## AUTHOR CONTRIBUTIONS

NJ was the principal investigator in this research. NJ and JMG led the writing of the paper. NJ, JMG, VG, JH, PGD, AJ, AK contributed to research concept and design of research tools. NJ, CM led the data analysis. All authors provided input into the final manuscript and its revised version.

## ETHICS STATEMENT

All potential ethics issues were considered during the design and implementation of this research. Ethics approval has been obtained for this project from the relevant Ethics Committee according to the regulations of the University of Cambridge.

## DATA ACCESSIBILITY STATEMENT

Certain data (excluding any personal data) can be made available upon request from the corresponding author.

## CONFLICT OF INTEREST

The authors declare no conflict of interest

## Supporting information


**TABLE S1** Sample characteristics
**TABLE S2** Results of principal component analysis
**TABLE S3** Descriptive statistics for the explanatory variables in the regression model
