# Supplementary material for: COVID‐19 and protected areas: Impacts, conflicts, and possible management solutions
Source: Conserv Lett. 2021 Apr 6;14(4):e12800. doi: 10.1111/conl.12800 (PMC8250896; doi:10.1111/conl.12800)
Supplement: Supplementary file 1 — TABLE S1 Sample characteristics TABLE S2 Results of principal component analysis TABLE S3 Descriptive statistics for the explanatory variables in the regression model [file CONL-14-0-s001.docx]

**Table S1** Sample characteristics

| **Age category** | **%** |
| --- | --- |
| 18-25 | 2.4 |
| 26-35 | 16.2 |
| 36-45 | 20.5 |
| 46-55 | 27.7 |
| 56-65 | 22.3 |
| 66-75 | 9.8 |
| 76-85 | 0.2 |
| 85+ | 0.2 |
| **Gender** | **%** |
| Male | 50% |
| Female | 50% |
| **Education** | **%** |
| Primary school level education completed/No formal qualifications completed | 1.1 |
| Entry level qualifications | 0.2 |
| Secondary education level qualifications (e.g. GCE O Level, CSE, GCSE, NVQ/BTEC Levels 1-2 | 6.3 |
| Sixth form level (e.g. A/AS Levels, NVQ/BTEC Level 3) | 10.9 |
| Further/ Higher education | 45.9 |
| Postgraduate degree (e.g. Masters, PhD. NVQ Level 5) | 35.6 |
| **Household Income** | **%** |
| No income | 2.6 |
| up to £25,000 | 23.3 |
| £25001- up to £50,000 | 35.9 |
| £50,001-£70,000 | 17.2 |
| over £70,000 | 9.6 |

**Table S2** Results of Principal Component Analysis

| **Factor** | **Variables** | **Component** | **Fit indices** |
| --- | --- | --- | --- |
| A1. Impact of reduced visitors numbers | Quiet walking paths | 0.891 | KMO Measure of sampling adequacy: 0.759  Cronbach’s alpha: 0.762 |
|  | Less busy cycling routes | 0.815 |  |
|  | Fewer visitors in beauty spots | 0.795 |  |
| A2. Impact on social interactions | Keep 2m distance | 0.794 |  |
|  | Shops closure | 0.755 |  |
|  | Socialise less often | 0.711 |  |
| A3. Impact of staying at home | Spend more time with household members | 0.729 |  |
|  | Less travel | 0.706 |  |
|  | Work from home | 0.650 |  |
| FACTOR B: Impact of restrictions on the use of the National Park | Quality of life | 0.931 | KMO Measure of sampling adequacy: 0.910  Cronbach’s alpha: 0.932 |
|  | Mental health | 0.918 |  |
|  | Connectedness to nature | 0.911 |  |
|  | Physical health | 0.889 |  |
|  | Recreation | 0.836 |  |
|  | Social relations | 0.707 |  |

**Table S3** Descriptive statistics for the explanatory variables in the regression model

| **Responses (%)** | **Factor A1** | | | **Factor A2** | | | **Factor A3** | | |
| --- | --- | --- | --- | --- | --- | --- | --- | --- | --- |
|  | **Quiet walking paths** | **Less busy cycling routes** | **Fewer visitors in beauty spots** | **Keep 2m distance** | **Shops closure** | **Socialise less often** | **Spend more time with household members** | **Less travel** | **Work from home** |
| Very negative | 3.6 | 2.8 | 6.8 | 9.9 | 11.4 | 28.5 | 5.4 | 10.2 | 8.3 |
| Negative | 5.6 | 6.1 | 10.5 | 39.6 | 36.4 | 52 | 6.6 | 17.2 | 12.4 |
| Neutral | 29 | 35.9 | 19.3 | 39.4 | 44.1 | 16.3 | 36.8 | 18 | 53.6 |
| Positive | 35.2 | 26.2 | 35.7 | 7.4 | 6 | 2.1 | 34.8 | 30.9 | 16.1 |
| Very Positive | 26.6 | 29 | 27.7 | 3.7 | 2.2 | 1.1 | 16.3 | 23.6 | 9.6 |
| TOTAL | 100 | 100 | 100 | 100 | 100 | 100 | 100 | 100 | 100 |
| **Responses (%)** | **Factor B** | | | | | | **The impact of lockdown on income level** | | |
|  | **Quality of life** | **Mental health** | **Connectedness to nature** | **Physical health** | **Recreation** | **Social relations** |  |  |  |
| Very negative | 17.5 | 19.1 | 14 | 13.6 | 39.4 | 9.7 | 26.3 | | |
| Negative | 35.5 | 29.2 | 27.7 | 32.1 | 27.6 | 14.6 | 19.4 | | |
| Neutral | 22.4 | 31.3 | 32.1 | 35.2 | 16.3 | 38.7 | 49.9 | | |
| Positive | 9.2 | 9.3 | 9.4 | 8.2 | 4.9 | 12.8 | 3.4 | | |
| Very Positive | 15.4 | 11.1 | 16.7 | 10.9 | 11.9 | 24.3 | 1.1 | | |
| TOTAL | 100 | 100 | 100 | 100 | 100 | 100 | 100 | | |
